# Rurality of patient residence and access to transplantation among children with kidney failure in the United States

**DOI:** 10.1007/s00467-023-06148-w

**Published:** 2023-09-28

**Authors:** Gabriela Accetta-Rojas, Charles E. McCulloch, Adrian M. Whelan, Timothy P. Copeland, Barbara A. Grimes, Elaine Ku

**Affiliations:** 1 Department of Epidemiology and Biostatistics, University of California, San Francisco, CA USA; 2 Department of Medicine, Division of Nephrology, University of California, 500 Parnassus Avenue MBU-E 414 SF, San Francisco, CA 94143-0532 USA; 3 Division of Nephrology, Department of Medicine, University of California, San Francisco, CA USA; 4 Division of Pediatric Nephrology, Department of Pediatrics, University of California, San Francisco, CA USA

**Keywords:** Rural, Pediatric transplantation, Living kidney donor, Deceased kidney donor

## Abstract

**Background:**

Residence in rural areas is often a barrier to health care access. To date, differences in access to kidney transplantation among children who reside in rural and micropolitan areas of the US have not been explored.

**Methods:**

A retrospective cohort study of children < 18 years who developed kidney failure between 2000 and 2019 according to the United States Renal Data System (USRDS). We examined the association between rurality of patient residence and time to living and/or deceased donor kidney transplantation (primary outcomes) and waitlist registration (secondary outcome) using Fine–Gray models.

**Results:**

We included 18,530 children, of whom 14,175 (76.5%) received a kidney transplant (39.8% from a living and 60.2% from a deceased donor). Residence in micropolitan (subhazard ratio (SHR) 1.16; 95% CI 1.06–1.27) and rural (SHR 1.18; 95% CI 1.06–1.3) areas was associated with better access to living donor transplantation compared with residence in metropolitan areas. There was no statistically significant association between residence in micropolitan (SHR, 0.95; 95%CI 0.88–1.03) and rural (SHR, 0.94; 95%CI 0.86–1.03) areas compared with metropolitan areas in the access of children to deceased donor transplantation. There was also no difference in the time to waitlist registration comparing micropolitan (SHR 1.04; 95%CI 0.98–1.10) and rural (SHR 1.05; 95% CI 0.98–1.13) versus metropolitan areas.

**Conclusions:**

In children with kidney failure, residence in rural and micropolitan areas was associated with better access to living donor transplantation and similar access to deceased donor transplantation compared with residence in metropolitan areas.

**Graphical abstract:**

**Figure:**
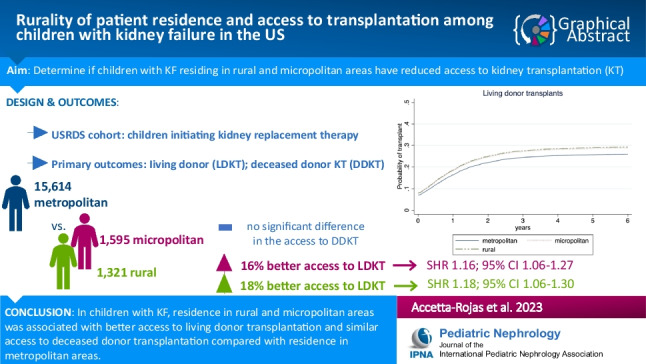
A higher resolution version of the Graphical abstract is available as Supplementary information

**Supplementary Information:**

The online version contains supplementary material available at 10.1007/s00467-023-06148-w.

## Introduction

The preferred modality of treatment for children with kidney failure (KF) is kidney transplantation. Between 2016 and 2020, more than 900 children were diagnosed with KF annually in the United States (US), but less than 200 pediatric kidney transplants were performed each year [1]. Considering that the number of donors is limited in the US, most children with KF will start dialysis as their initial treatment modality, which may be associated with a high burden of cardiovascular risk factors and neurodevelopmental complications [2]. Despite their higher priority on the kidney transplant waitlist (compared to adults), children in the US also face barriers in access to timely kidney transplantation [3, 4].

In both nephrology and disciplines outside of nephrology, residence in rural areas of the US has been shown to pose as a barrier to quality health care [5]. Rural and micropolitan counties represent the majority of the health professional shortage areas in the US [6], have higher child poverty rates [7], and have worse observed health outcomes [8–10]. In adults with KF, studies of the association between residence in rural areas and access to kidney transplantation have been inconsistent in their findings, with some studies showing the presence of an association and others showing a lack thereof [11, 12]. To date, differences in the access to kidney transplantation of children who reside in rural and micropolitan areas of the US to kidney transplantation have not been explored.

The primary objective of this study was to determine whether children with KF living in rural areas have differential access to kidney transplantation or waitlisting. We hypothesized that residence in rural areas would be associated with longer time to waitlisting and kidney transplantation in children with KF.

## Methods

### Study population and data source

We performed a retrospective cohort study using data from the United States Renal Data System (USRDS), which is the national registry of all patients treated with dialysis or kidney transplantation in the US. Children with KF aged 0 to 17 years old who started kidney replacement therapy (KRT) from January 1, 2000, to December 31, 2019, were included in the study. Children with missing covariates and living in US territories were excluded.

Demographic characteristics were extracted from Patients file and the Centers for Medicare and Medicaid Services Medical Evidence 2728 (MEDEVID) form at the time of KF onset. Race/ethnicity was based on provider attestation in the Patients file. Race/ethnicity was categorized as Hispanic, Black, non-Hispanic White, and Other.

This study was reviewed by the University of California San Francisco Institutional Review Board and considered to be exempt human subjects research.

### Primary predictor

Rurality of the patient residence was determined using the rural–urban commuting area (RUCA) codes as defined by the United States Department of Agriculture. Zip codes of residence were matched to RUCA codes which ranged from 1.0 (most urban) to 10.3 (most rural) based on population size and commuting flow [13]. We categorized each patient’s residence at the start of KRT as metropolitan (1.0–3.9, corresponding to urbanized areas with ≥ 50,000 population); micropolitan (4.0–6.0, corresponding to urban clusters of 10,000–49,999 population); or small town/rural areas (7.0–10.3, towns with population of lower than 10,000 inhabitants and outside urban areas and urban clusters) [14].

### Outcomes

The primary outcome was time to kidney transplantation starting from the date of dialysis initiation. If patients received preemptive transplantation, time to kidney transplantation was set at 0.5 days. We restricted our analyses to only the first kidney transplant event. We then examined outcomes separately by whether the donor source was living or deceased.

Our secondary outcome was time to waitlist registration starting from the date of dialysis initiation. If a patient was preemptively waitlisted, time to waitlist registration was set at 0.5 days.

### Statistical analysis

The association between rurality of the patient residence and time to transplantation or waitlisting was examined using separate Fine–Gray subhazard models for each outcome and treating death as a competing risk. Patients were censored administratively on December 31, 2019. The model was adjusted for age, sex, race/ethnicity, primary cause of KF, region of the US (Northeast, South, Midwest, and West), calendar year of onset of KF (grouped in 5-year categories) health insurance status (Medicare/Medicaid, private, or none), and income as the neighborhood median income by zip code of patient’s residence [15]. We did not adjust for comorbidities as the prevalence of comorbidities (e.g., heart failure) in children is low [16]. The proportional hazards assumption was tested with Schoenfeld residuals and log–log plot.

The association between rurality of patient residence and time to deceased donor kidney transplantation was examined using Fine–Gray models treating death and living donor kidney transplantation as competing risks. When living donor kidney transplantation was considered the outcome of interest, death and deceased donor transplantation were considered competing risks.

We assessed for interactions between rurality of patient residence and race/ethnicity, neighborhood median income, calendar year, and region of US as defined a priori. Interactions were considered statistically significant if the *p* value was < 0.05. All analyses were conducted using Stata 17 (StataCorp, College Station, TX).

## Results

### Study participants

A total of 18,530 children started KRT during the study period and were included for analysis. We excluded 1282 patients: 959 with a missing MEDEVID form or missing covariates and 325 for residing in US territories. We tested for differences in the proportion of children excluded due to missing covariates (*N* = 1282) by rurality of residence (if zip code was available, *N* = 822) and did not find any difference (*p* = 0.06).

A total of 15,614 (84.3%) of the children included for analysis resided in metropolitan areas, whereas only 1595 (9%) resided in micropolitan areas and 1321 (7%) in rural areas at time of KF onset. The age at dialysis initiation was similar across metropolitan, micropolitan, and rural areas (Table 1). Among children living in rural areas, most were non-Hispanic White (68.2%). The primary causes of KF among the three groups were similar, with urologic related causes being slightly more common in rural (19.1%) versus micropolitan (15.8%) and metropolitan (15.8%) areas. Differences were also noted in dialysis modality at time of initiation of KRT: a higher proportion of peritoneal dialysis was used in rural (44%) and micropolitan (45%) versus metropolitan (37%) areas.

**Table 1:** Characteristics of patients by rurality of residence

	Metropolitan	Micropolitan	Rural
	*n* = 15,614	*n* = 1595	*n* = 1321
Age at incidence of KF (years), *n* (%)			
0–5	4358 (27.9)	443 (27.8)	378 (28.6)
6–11	3279 (21.0)	314 (19.7)	288 (21.8)
12–17	7977 (51.1)	838 (52.5)	655 (49.6)
Male	8903 (57.0)	903 (56.6)	731 (55.3)
Race and ethnicity, *n* (%)			
Black	3619 (23.2)	274 (17.2)	178 (13.5)
Hispanic	4354 (27.9)	270 (16.9)	152 (11.5)
Non-Hispanic White	6656 (42.6)	985 (61.8)	901 (68.2)
Other	985 (6.3)	66 (4.1)	90 (6.8)
Health insurance status, *n* (%)			
None	729 (4.7)	68 (4.3)	55 (4.2)
Medicare/Medicaid	6764 (43.3)	822 (51.5)	699 (52.9)
Private	8121 (52.0)	705 (44.2)	567 (42.9)
Region of US, *n* (%)			
West	3974 (25.5)	296 (18.6)	192 (14.5)
Midwest	3057 (19.6)	405 (25.4)	467 (35.4)
South	6007 (38.5)	763 (47.8)	556 (42.1)
Northeast	2576 (16.5)	131 (8.2)	106 (8.0)
Primary cause of KF, *n* (%)			
Glomerulonephritis	4773 (30.6)	479 (30.0)	355 (26.9)
Other cause	4294 (27.5)	452 (28.3)	401 (30.4)
Urologic	2471 (15.8)	252 (15.8)	254 (19.2)
Cystic kidney	1813 (11.6)	193 (12.1)	143 (10.8)
Unknown	1334 (8.5)	132 (8.3)	97 (7.3)
Hypertension	591 (3.8)	56 (3.5)	42 (3.2)
Diabetes	338 (2.2)	31 (1.9)	29 (2.2)
Modality, *n* (%)			
Hemodialysis	7228 (46.3)	599 (37.6)	474 (35.9)
Peritoneal dialysis	5775 (37.0)	718 (45.0)	581 (44.0)
Neighborhood income, $1000, median (IQR)	52 (40.74–67.03)	41.19(36.03–46.44)	39.3(33.8–45.17)
Era of KF onset, *n* (%)			
2000–2004	3907 (25.0)	422 (26.5)	338 (25.6)
2005–2009	4192 (26.8)	430 (27.0)	363 (27.5)
2010–2014	3875 (24.8)	394 (24.7)	333 (25.2)
2015–2019	3640 (23.3)	349 (21.9)	287 (21.7)

*IQR* interquartile range

*KF* kidney failure

### Access to kidney transplantation

The median follow-up between dialysis initiation and kidney transplantation was 1.36 (IQR, 0.68–2.4) years, during which 14,175 (76.5%) children received a kidney transplant (39.8% from living donors and 60.2% from deceased donors). The adjusted subhazard ratio for time to kidney transplantation from any donor source was 1.06 (95% CI 0.99–1.14) for children living in rural areas and 1.06 (95% CI 0.99–1.13) for children living in micropolitan areas compared with children living in metropolitan areas.

**Fig. 1:**
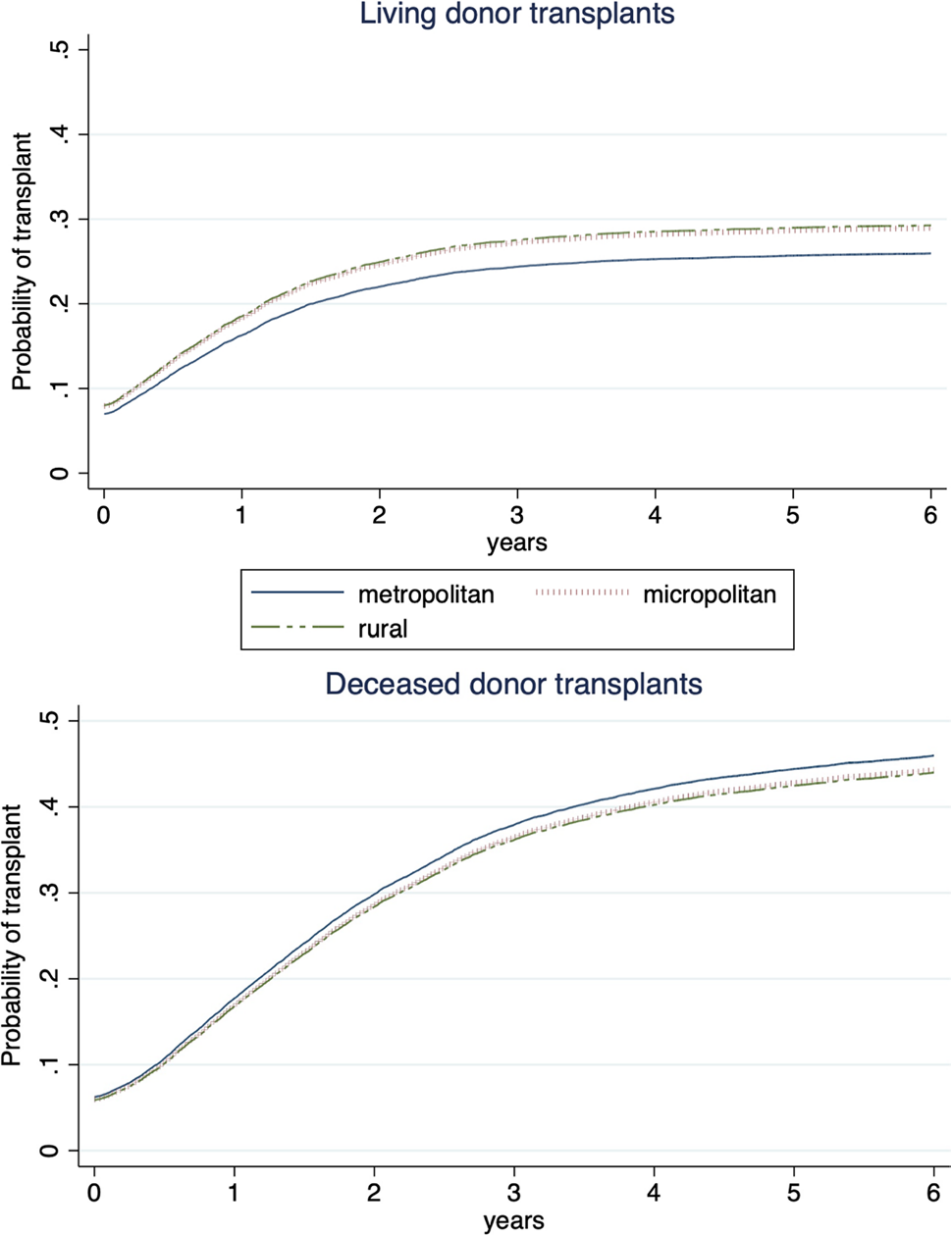
Cumulative incidence function for transplantation rural and micropolitan vs. metropolitan residence, accounting for competing risks. Adjusted for age at kidney failure onset, sex, primary cause of kidney failure, race/ethnicity, median neighborhood income, health insurance status, region of US, and year of kidney failure onset. Time to preemptive transplantation was set at 0.5 days

When considering living donor transplantation as the outcome of interest and treating deceased donor transplantation and death as competing events, residence in rural (SHR 1.18; 95% CI 1.06–1.30) and micropolitan areas (SHR 1.16; 95% CI 1.06–1.27) was associated with higher subhazard of living donor transplantation compared to residence in metropolitan areas in adjusted analyses. Alternatively, when considering deceased donor transplantation as the outcome of interest, there was no difference in the risk comparing residence in rural (SHR 0.94; 95% CI 0.86–1.03) and micropolitan (SHR 0.95; 95% CI 0.88–1.03) areas compared with metropolitan areas (Fig. 1 and Table 2).

**Table 2:** Subhazard ratio (95% CI) for living donor transplantation, deceased donor transplantation and waitlist registration

	Rurality of residence		
	Metropolitan (reference)	Micropolitan	Rural
Kidney transplant			
Unadjusted	1	1.03 (0.95–1.07)	1.04 (0.98–1.11)
Adjusted	1	1.06 (0.99–1.13)	1.06 (0.99–1.14)
Living donor transplant			
Unadjusted	1	1.09 (1.00–1.19)	1.18 (1.07–1.29)
Adjusted	1	1.16 (1.06–1.27)	1.18 (1.06–1.30)
Deceased donor transplant			
Unadjusted	1	0.94 (0.87–1.01)	0.90 (0.82–0.98)
Adjusted	1	0.95 (0.88–1.03)	0.94 (0.86–1.03)
Waitlist registration			
Unadjusted	1	0.98 (0.92–1.03)	1.01 (0.94–1.07)
Adjusted	1	1.04 (0.98–1.10)	1.05 (0.98–1.13)

Adjusted for age at kidney failure onset, sex, primary cause of kidney failure, race/ethnicity, median neighborhood income, health insurance status, region of US, and year of kidney failure onset

The association between rurality of patient residence and deceased donor transplantation differed by race/ethnicity (*p* value for interaction = 0.032). After stratification according to race/ethnicity, children of Other race/ethnicity (Alaska Native, American Indian, Arabian, Asian, Middle Eastern, Native Hawaiian and Pacific Islander, or those with unknown race) had lower access to deceased donor kidney transplantation if they resided in micropolitan areas versus metropolitan areas (Supplementary Table 1).

No statistically significant interaction was identified between rurality of patient residence and race/ethnicity in terms of access to living donor transplantation. In addition, no statistically significant interaction was identified between rurality of patient residence and calendar year, region of US, or neighborhood median income in terms of access to either living or deceased donor transplantation (all *p* values for interaction > 0.05).

### Waitlist registration

A total of 15,187 children (82%) in the cohort were registered on the kidney transplant waiting list, of whom 4163 (27.4%) were preemptively waitlisted. Among those who were not preemptively waitlisted, the median time between dialysis initiation and waitlist registration was 1.08 (IQR, 0.47–2.4) years. The adjusted subhazard ratio for waitlist registration was 1.05 (95% CI 0.98–1.13) for those living in rural areas and 1.04 (95% CI 0.98–1.10) for those living in micropolitan areas compared with those living in metropolitan areas (Table 2). No statistically significant interaction was noted between rurality of patient residence and race/ethnicity, calendar year, region of US, or neighborhood median income (all *p* values for interaction > 0.05).

## Discussion

In this national cohort of children starting KRT, we observed that children living in rural and micropolitan areas had better access to living donor transplantation in comparison to those living in metropolitan areas. In contrast, there was no difference in access to deceased donor transplant or waitlisting by rurality of patient residence.

A recent study on the association between rurality of residence and kidney transplantation access in adults demonstrated a higher likelihood of transplantation among those residing in rural and micropolitan areas compared with metropolitan areas [12]. This is consistent with the 18% better access of children residing in rural areas and 16% better access of children residing in micropolitan areas to living donor transplantation compared with children residing in metropolitan areas. We speculate that families living in rural and micropolitan areas may have higher motivation to donate and/or seek living donor transplantation given the longer travel distance to the nearest pediatric dialysis unit. Alternatively, it is possible that those who reside in rural areas might have stronger social bonds and higher levels of community support from family and friends compared with those residing in metropolitan areas [17, 18], and consequently, a higher chance of identifying a donor. This would be consistent with findings in an adult cohort of veterans, where those residing in rural areas had a higher proportion of living donors and higher proportion of non-biologically related living donors compared with those residing in metropolitan areas [19].

In children, the lack of an association between rurality of residence and access to deceased donor transplantation could be explained by their overall priority for deceased donor organs within the kidney allocation system, which could circumvent any delays that may have been encountered during the diagnosis and referral of children to a transplant center for waitlisting. Although rural areas are known to have greater shortages of pediatric health care professionals and the number of pediatric nephrologists is declining overall in the US [20], it is reassuring that there were no differences that we could identify in time to waitlisting and transplantation in children by rurality of residence. This is also consistent with prior studies which have suggested that distance between a children’s residence and transplant centers was not associated with differential access to kidney transplantation [21].

We observed that among children of other race/ethnicity, those living in micropolitan areas have worse access to deceased donor transplantation when compared to those living in metropolitan areas. The reasons for these findings are unclear, especially since this group encompasses children of more than one racial group, but reasons for this observation should be explored further.

The main strengths of this study include the large sample size of children included for study and use of data that are nationally representative. However, we note that our findings may differ from those in other nations since the characteristics of individuals residing in rural regions [22] and geographic distribution of health care resources may vary widely in different nations. In addition, the definition of rurality may also vary between countries which may limit the generalizability of our findings. The definition used by the US Department of Agriculture is similar to that used in the United Kingdom [23], where areas with fewer than 10,000 inhabitants are considered to be rural. Consistent with our observations, children living in non-rural areas in the United Kingdom also were not observed to have better access to preemptive kidney transplantation after adjusting for social deprivation [24]. In contrast, in Canada and Australia, both population size and the population per km^2^ and remoteness are considered in the definition of rurality. Contrary to our findings, children living in remote areas of Australia were 35% less likely to receive a preemptive living donor kidney transplantation when compared to those living in metropolitan areas [25].

A few other limitations of our study should be noted. We are unable to account for changes in patient residence after onset of KF; however, notwithstanding this limitation, the proportion of those moving to metropolitan areas was estimated to be low in the adult population [12] and would likely result in a bias toward the null. We also do not have granular data on whether some living donors were available or deemed ineligible by transplant centers, as donor data are not captured in the USRDS if they did not undergo nephrectomy.

In conclusion, we observed modestly better access of children with KF living in rural and micropolitan areas (vs. metropolitan areas) to living donor kidney transplantation, but not to deceased kidney transplantation or waitlist registration. Further studies are needed to understand how to optimize access of children to kidney transplantation, regardless of their geographic location.

### Supplementary Information

Below is the link to the electronic supplementary material.

Graphical abstract (PPTX 758 KB)

Supplementary file1 (DOCX 14.5 KB)

## Data Availability

The data in this manuscript are publicly available through the US Renal Data System.

## Declarations

### Disclaimer

The interpretation and reporting of these data are the responsibility of the authors and in no way should be seen as an official policy or interpretation of the US government.
