# Supplementary material for: Rurality of patient residence and access to transplantation among children with kidney failure in the United States
Source: Pediatr Nephrol. 2023 Sep 28;39(4):1239–44. doi: 10.1007/s00467-023-06148-w (PMC10899312; doi:10.1007/s00467-023-06148-w)
Supplement: Supplementary file 1 — Graphical abstract (PPTX 758 KB) [file 467_2023_6148_MOESM1_ESM.pptx]

## Slide 1
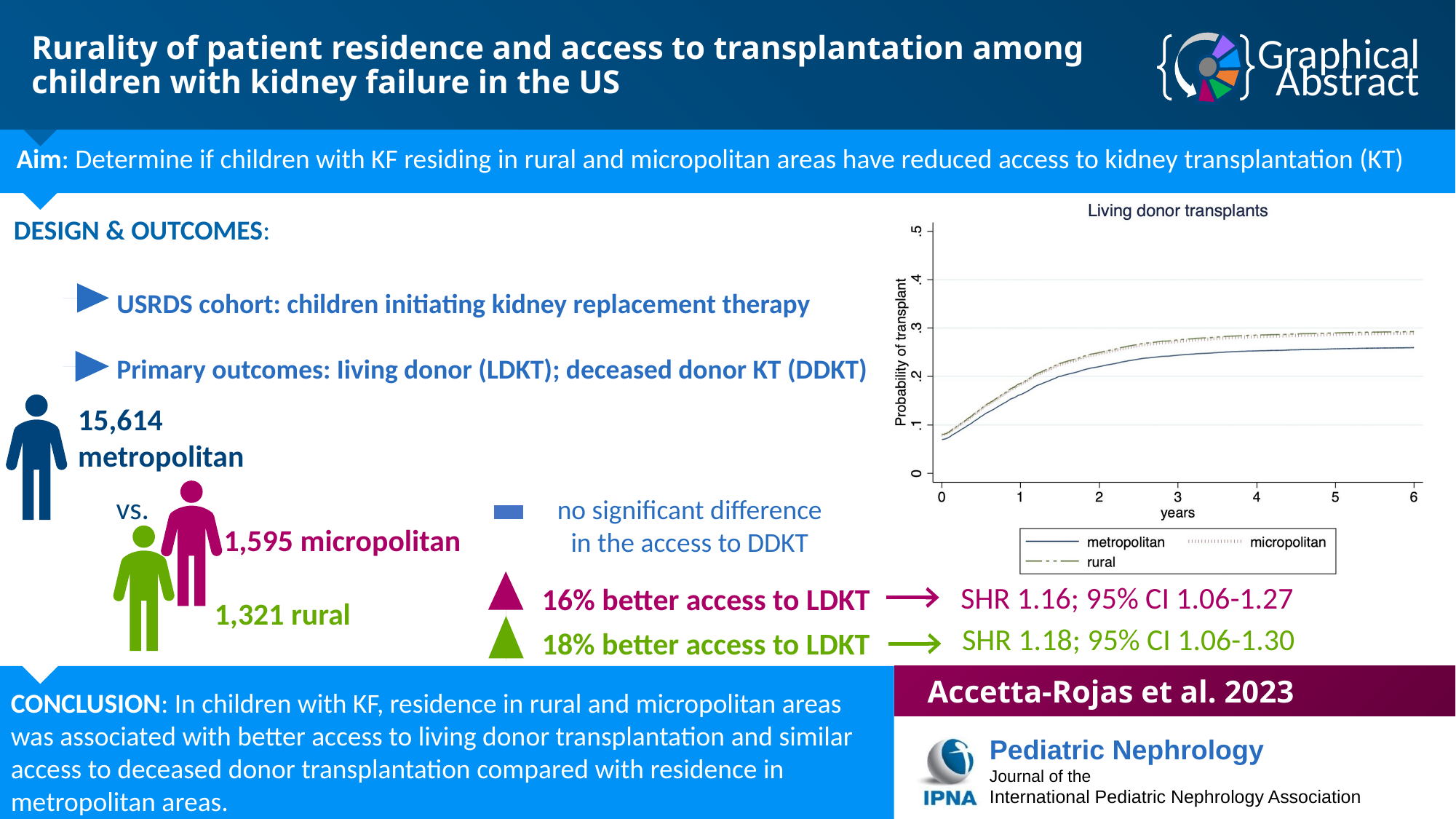

Rurality of patient residence and access to transplantation among
children with kidney failure in the US
Aim: Determine if children with KF residing in rural and micropolitan areas have reduced access to kidney transplantation (KT)
DESIGN & OUTCOMES:
USRDS cohort: children initiating kidney replacement therapy
Primary outcomes: Iiving donor (LDKT); deceased donor KT (DDKT)
15,614 metropolitan
vs.
no significant difference in the access to DDKT
1,595 micropolitan
SHR 1.16; 95% CI 1.06-1.27
16% better access to LDKT
1,321 rural
SHR 1.18; 95% CI 1.06-1.30
18% better access to LDKT
Accetta-Rojas et al. 2023
CONCLUSION: In children with KF, residence in rural and micropolitan areas was associated with better access to living donor transplantation and similar access to deceased donor transplantation compared with residence in metropolitan areas.
