# Supplementary material for: Rurality of patient residence and access to transplantation among children with kidney failure in the United States
Source: Pediatr Nephrol. 2023 Sep 28;39(4):1239–44. doi: 10.1007/s00467-023-06148-w (PMC10899312; doi:10.1007/s00467-023-06148-w)
Supplement: Supplementary file 2 — Supplementary file1 (DOCX 14.5 KB) [file 467_2023_6148_MOESM2_ESM.docx]

**Supplementary Table 1.** Subhazard ratio (95% CI) for deceased donor transplantation stratified according to race/ethnicity

| Race/ethnicity | **Metropolitan (reference)** | | **Micropolitan** | **Rural** |
| --- | --- | --- | --- | --- |
| Black | 1 | 0.99 (0.83-1.19) | | 0.90 (0.72-1.44) |
| Hispanic | 1 | 1.01 (0.86-1.88) | | 0.98 (0.80-1.22) |
| Non-Hispanic White | 1 | 0.92 (0.82-1.03) | | 0.89 (0.79-1.00) |
| Other | 1 | 0.56 (0.36-0.85) | | 0.98 (0.71-1.34) |

Model adjusted for age at kidney failure onset, sex, primary cause of kidney failure, median neighborhood income, health insurance status, region of US, year of kidney failure onset

P value for interaction between race/ethnicity and area of residence = 0.032
